# A case of chronic gastric anisakiasis coexisting with early gastric cancer

**DOI:** 10.20407/fmj.2022-010

**Published:** 2022-10-28

**Authors:** Eiko Sakurai, Masaaki Okubo, Yutaka Tsutsumi, Tomoyuki Shibata, Tomomitsu Tahara, Yuka Kiriyama, Ayano Michiba, Naoki Ohmiya, Tetsuya Tsukamoto

**Affiliations:** 1 Department of Diagnostic Pathology, Fujita Health University, Graduate School of Medicine, Toyoake, Aichi, Japan; 2 Department of Gastroenterology, Fujita Health University, Graduate School of Medicine, Toyoake, Aichi, Japan; 3 Diagnostic Pathology Clinic, Pathos Tsutsumi, Inazawa, Aichi, Japan

**Keywords:** Nematode, Anisakiasis, Stomach, Gastric cancer

## Abstract

**Background:**

Anisakiasis is a parasitic disease caused by the consumption of raw or undercooked fish that is infected with *Anisakis* third-stage larvae. In countries, such as Japan, Italy, and Spain, where people have a custom of eating raw or marinated fish, anisakiasis is a common infection. Although anisakiasis has been reported in the gastrointestinal tract in several countries, reports of anisakiasis accompanied by cancer are rare.

**Case presentation:**

We present the rare case of a 40-year-old male patient with anisakiasis coexisting with mucosal gastric cancer. Submucosal gastric cancer was suspected on gastric endoscopy and endoscopic ultrasonography. After laparoscopic distal gastrectomy, granulomatous inflammation with *Anisakis* larvae in the submucosa was pathologically revealed beneath mucosal tubular adenocarcinoma. Histological and immunohistochemical investigation showed cancer cells as intestinal absorptive-type cells that did not produce mucin.

**Conclusion:**

*Anisakis* larvae could have invaded the cancer cells selectively because of the lack of mucin in the cancerous epithelium. Anisakiasis coexisting with cancer is considered reasonable rather than coincidental. In cancer with anisakiasis, preoperative diagnosis may be difficult because anisakiasis leads to morphological changes in the cancer.

## Introduction

Health hazards resulting from the ingestion of raw food have distressed people across the world since ancient times. Anisakiasis is a type of food poisoning caused by the ingestion of raw fish infected with larval nematodes of the *Anisakidae* family. Approximately 20,000 cases of anisakiasis are reported in the world annually, and >90% are from Japan.^1^ Recently, the consumption of undercooked fish has become popular in other countries; thus, anisakiasis is becoming a global disease. The recent development of transport systems that can deliver fresher fish have increased the prevalence of anisakiasis. In 1960, van Thiel et al.^2^ first described that this disease was caused by the *Anisakis* larvae, in the Netherlands.

There are three types of *Anisakis* larvae: *Anisakis simplex* (*Anisakis* larvae type I), *Anisakis physeteris* (*Anisakis* larvae type II), and *Pseudoterranova decipiens*. Among the three types of larvae, *Anisakis simplex* is the most common pathogen in anisakiasis.

The larvae of *Anisakis* change their form four times (L1–L3, and the adult worm) in their life cycle. The adult worm lives in marine mammals (final host), such as whales, seals, and dolphins.^3^ Eggs are produced in the final host and excreted by the mammal into seawater. These first-stage larvae (L1) become embryonated eggs and hatch into free-swimming larvae—the second-stage larvae (L2). Small crustaceans (first intermediate host) eat the L2 larvae, which develop into third-stage larvae (L3). Infected crustaceans are then ingested by fish and squid (second intermediate host). The L3 larvae are transferred from smaller to larger fish, and finally, marine mammals eat infected fish, and the L3 larvae mature into adult worms. If humans consume infected raw or undercooked fish, they can become incidental hosts. The L3 larvae enter the digestive duct and cause anisakiasis. The length of *Anisakis* third-stage larvae is 20–30 mm.

The following four types of disease are caused by *Anisakis* larvae: gastric anisakiasis, intestinal anisakiasis, extraintestinal anisakiasis, and *Anisakis* allergy. Gastric anisakiasis is the most common form, which is caused by direct larval invasion into the gastric mucosa and causes abdominal pain, nausea, and vomiting. The easy availability of endoscopy has enabled the identification of a large number of cases of gastric anisakiasis. Intestinal anisakiasis sometimes leads to extraintestinal anisakiasis if the larvae penetrate the intestinal wall. *Anisakis* allergy was first reported in 1990 in Japan,^4^ and there are various symptoms, such as urticaria, digestive symptoms (diarrhea, vomiting, or abdominal pain), and anaphylactic shock. Allergen-specific immunoglobulin E detection is useful for establishing a diagnosis of *Anisakis* allergy.

The symptoms of anisakiasis are classified as acute and chronic anisakiasis. Patients with acute anisakiasis exhibit serious symptoms, such as severe abdominal pain, nausea, and vomiting. Chronic anisakiasis is asymptomatic or causes mild intermittent abdominal pain for several weeks. If patients have been previously sensitized to *Anisakis* antigens, acute and drastic symptoms occur as a result of an immediate allergic response. Chronic anisakiasis occurs in never-sensitized patients and presents as a foreign-body reaction in the pathological findings (Table 1).^5–7^

Gastric cancer is the fifth most common and fourth most lethal cancer globally.^8^ The cause of gastric cancer, the most common histopathological type being adenocarcinoma, is multifactorial, although infection with *Helicobacter pylori* is a major risk factor for gastric cancer.^9^ Early gastric cancer is treated with endoscopic resection, whereas the main treatment for non-early operable gastric cancer is surgery.

Although gastric anisakiasis and gastric cancer are common diseases, cases with both diseases coexisting have been reported rarely. It is uncertain whether gastric cancer may provide a beneficial environment for the *Anisakis* larvae or whether both lesions exist coincidentally.

Here, we describe the case of a patient with anisakiasis coexisting with early gastric cancer.

## Case Presentation

A 40-year-old man with a gastric ulcer, which was diagnosed owing to a suspicion of gastric cancer during screening, was referred to our hospital for further diagnostic evaluation.

The patient had a history of a stomach ulcer and reflux esophagitis. *Helicobacter pylori* eradication was performed 1 year before cancer screening. He expressed no complaints during the medical examination in our hospital.

**Figure 1:**
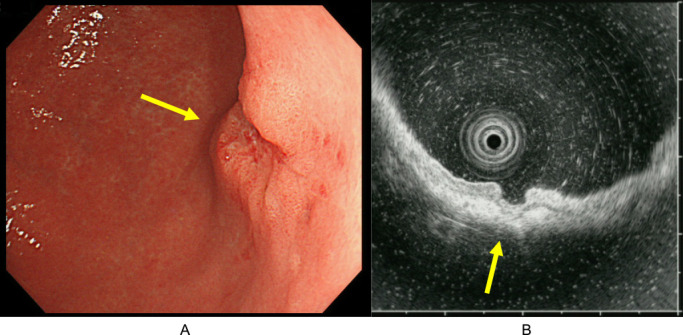
Gastric endoscopy and endoscopic ultrasonography findings. (A) Endoscopy revealed superficial adenocarcinoma (arrow) on the posterior wall in the corpus of the stomach. (B) In the endoscopic ultrasonography, the tumor was suspected to have invaded the second layer (mucosa) with partial invasion into the third layer (submucosa) (arrow).

Gastrointestinal endoscopy (Figure 1) revealed a superficial ulcer on the posterior wall of the corpus of the stomach. Irregular microvascular and microsurface patterns and a demarcation line on a superficial depression were seen using magnifying endoscopy with narrow-band imaging. The lesion was diagnosed as early gastric cancer, and invasion to the submucosa was suspected on endoscopic ultrasonography.

Contrast-enhanced computed tomography could not clearly identify the lesion in the stomach or metastatic lesions.

The results of a complete blood count and tumor marker evaluation were almost normal, including the eosinophil count (Table 2).

The tentative diagnosis was early gastric cancer with invasion into the submucosa. No metastasis to lymph nodes or other organs was suspected in the clinical findings. Laparoscopic distal gastrectomy was then performed.

**Figure 2:**
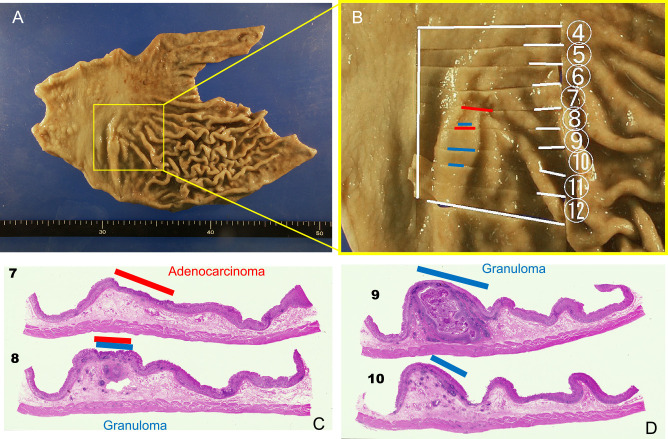
Macroscopic and loupe view. (A and B) Macroscopic view. The yellow square in A is enlarged in panel B. A shallow depression is visible. (C and D) A submucosal granuloma (blue line) beneath the intramucosal adenocarcinoma (red line) is seen in the loupe view.

**Figure 3:**
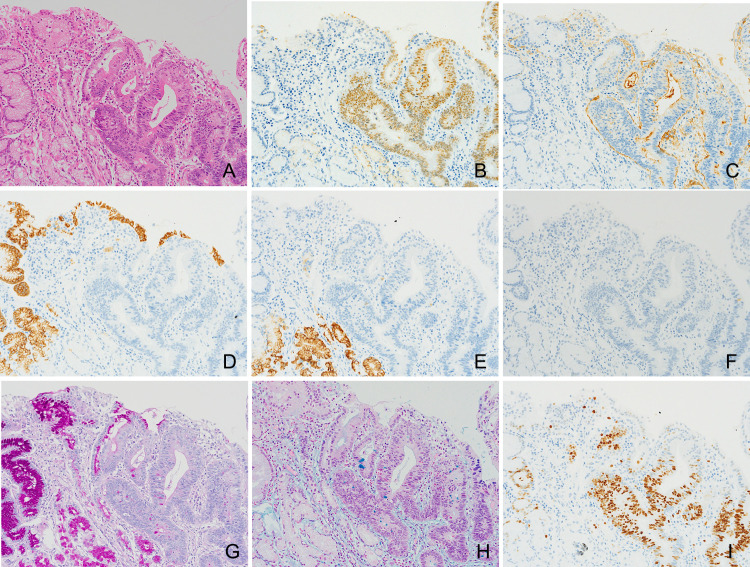
Histological and immunohistochemical features of the adenocarcinoma in the mucosa. (A) Hematoxylin and eosin staining. (B–F) Immunohistochemistry for cancer phenotype. Intestinal absorptive markers, CD10 (B) and CDX-2 (C). Mucin core proteins (MUCs), MUC5AC (D), MUC6 (E), and MUC2 (F). (G and H) Mucin histochemistry. Cancer cells were negative for mucin with PAS (G) and Alcian blue (H) staining. Proliferative marker, Ki-67 (I). Original magnification, ×200 (A–I). CD10, cluster of differentiation 10; CDX-2, caudal type homeobox 2; PAS, periodic acid-Schiff.

**Figure 4:**
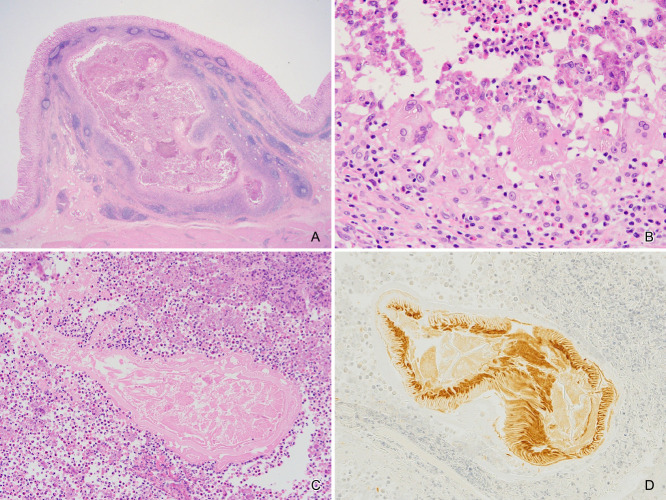
Granuloma. The submucosal lesion in (A) was a granuloma accompanied by necrosis, severe neutrophil and macrophage infiltration, and the presence of giant cells (B). Foreign material is surrounded by a granuloma in (C) and was proven to be *Anisakis* larvae immunohistochemically (D). Original magnification, ×12.5 (A) and ×200 (B–D).

A shallow depression was seen on the gastric mucosa in the macroscopic view (Figure 2). Histologically, gastric cancer (well-differentiated tubular adenocarcinoma) was confirmed in the mucosa, without submucosal invasion (Figures 2 and 3A). The fact that the cancerous epithelium was positive for caudal type homeobox 2 (Figure 3B) and cluster of differentiation 10 (Figure 3C) proved that the epithelium was intestinal absorptive epithelium. Mucin core protein (MUC) in the cancer cells was negative for MUC5AC (Figure 3D), MUC6 (Figure 3E), and MUC2 (Figure 3F). Periodic acid-Schiff (PAS) (Figure 3G) and Alcian blue (Figure 3H) staining were also negative, showing that the cancer cells lacked mucin. Ki-67 was irregularly distributed (Figure 3I). Beneath the cancer, granulomas were present in the submucosal lesion (Figure 4A). Necrosis, severe neutrophil and macrophage infiltration, and giant cells were observed in the granulomas (Figure 4B). There were few eosinophils in these areas. Foreign material surrounded by the granulomas was suspected to indicate the *Anisakis* larval digestive tract (Figure 4C). Immunohistologically, these lesions proved to be chronic gastric *Anisakis* granulomas (Figure 4D).^10^

Considering these findings, the cancerous lesion was confirmed as intramucosal gastric adenocarcinoma, which coexisted with submucosal granulomas caused by chronic anisakiasis.

## Discussion

In this case, we recognized two important issues about cancer coexisting with anisakiasis. First, *Anisakis* larvae might invade cancerous epithelium selectively. Our immunohistochemical investigation suggested that changes in mucin production in cancer cells could allow the *Anisakis* larvae to easily invade the gastric epithelium. Second, cancer accompanied by anisakiasis is troublesome for gastroenterologists because in this situation, it might be difficult to make a correct clinical diagnosis before operation. Anisakiasis leads to morphological changes in cancer tissue and an unclear depth of invasion, resulting in an incorrect clinical diagnosis of the cancer depth.

Although there have been reports of cancer with anisakiasis from several countries, most cases have been from Japan. Sonoda et al.^11^ discussed the details of 29 patients with anisakiasis associated with gastrointestinal cancer in Japan over a 43-year period (1970–2013). In our literature search, we identified 42 cases of cancer coexisting with anisakiasis reported from 1966 to 2021, including our case (Table 3);^5,6,11–48^ 38 cases were reported from Japan. Of the 42 cases, there were 36 reports of gastric cancer with anisakiasis, with 1 report of anisakiasis with duodenal cancer and 5 reports of anisakiasis with colon cancer. Twenty-five cases comprised cancers attached directly to the anisakiasis lesions, whereas other 17 cancers existed separately from the anisakiasis lesions. Among 17 cases, 7 gastric cancers with gastric anisakiasis lesions were located away from each other. In one case,^37^ colonic anisakiasis was revealed by colonoscopy prior to operation for gastric cancer. Yoo et al.^39^ overdiagnosed coexisting lesions of colon cancer and colonic anisakiasis as double primary cancer preoperatively. In seven cases,^23–25,30–32,41^ lymph nodes affected by *Anisakis* were observed postoperatively, and in four cases,^24,30,31,41^ enlarged lymph nodes around the cancer were overdiagnosed as metastasis. Three cases (24, 27, and 31) of anisakiasis were overdiagnosed as dissemination of gastric cancer, and three cases (13, 16, 19) of cancer attached directly to the anisakiasis lesions were underdiagnosed as a submucosal tumor or as an ulcer.

Clinical cancer depth was misjudged in six cases^26,33,43,46,47^ including in our case of gastric cancer attached directly to the anisakiasis lesions. In all six cases, the clinical diagnosis of cancer depth was deeper than that of the pathological diagnosis, resulting in overdiagnosis.

In summary, among the 42 cases, there were 12 cases of overdiagnosis and 3 cases of underdiagnosis. Sato et al.,^37^ Nakagawa et al.,^43^ and Sai et al.^47^ reported difficulty in obtaining a preoperative diagnosis in cases of inflammation or granulomas formed by anisakiasis just below the cancer. In our case, the preoperative clinical diagnosis was that of a submucosal cancerous lesion, and postoperatively, the pathological findings revealed that the lesion was mucosal gastric cancer with submucosal granulomatous inflammation accompanied by anisakiasis.

Various histological types of cancer have been reported in cases of cancer with anisakiasis. Twenty-three previous cases were diagnosed as differentiated carcinoma; tubular adenocarcinoma was diagnosed in 19 cases, and papillary carcinoma was diagnosed in 4 cases. Signet cell carcinoma and poorly differentiated adenocarcinoma were diagnosed in nine and two cases, respectively. In most cases, pathological stage I was diagnosed.

In cases of anisakiasis without cancer, it is also important to recognize that anisakiasis might lead to an incorrect clinical diagnosis. Murata et al.^49^ reported a patient with anisakiasis in the liver that mimicked metastatic liver cancer. The authors stated that inflammatory granulomas in chronic anisakiasis might be confused with other granulomatous diseases, such as gastrointestinal tuberculosis, sarcoidosis, and Crohn’s disease.^5^ A submucosal lesion associated with an *Anisakis* larva could be misdiagnosed as a submucosal tumor.^50^ Submucosal granulomas also misled us regarding the preoperative depth of invasion in our case.^16^ Yanagishita^51^ experienced a case of gastric anisakiasis that implied the possibility of latent linitis plastica type gastric cancer because the cancer induced limited swelling of the gastric fold.

Although some reports have shown that the coexistence of *Anisakis* larvae and cancer is not coincidental,^6,11,13,16,18,33^ only two reports have discussed a relationship between the change in mucin production in cancer cells and the tendency for *Anisakis* larvae to invade the cancer. Tsutsumi and Fujimoto^6^ showed a decrease in mucin production in cancerous epithelium using Alcian blue and PAS staining and stated that the change in mucin production allowed the *Anisakis* larvae to easily invade the gastric mucosa. In our case, in addition to Alcian blue and PAS staining, immunohistochemical investigation was performed. Our findings also suggest that *Anisakis* larvae may easily invade abnormal gastric mucosa that produces less mucin than that of normal gastric mucosa.

There are several reports in which *Anisakis* larvae were present near gastric ulcers. Ino et al.^52^ showed that *Anisakis* larvae gathered around an artificial gastric ulcer and implied that the ulcer and cancerous lesions helped the *Anisakis* larvae penetrate the mucosa. Kasuga et al.^53^ reported a case of anisakiasis in which *Anisakis* larvae invaded a gastric ulcer after endoscopic submucosal dissection for early gastric cancer. These cases support our suggestion because ulcerous epithelium is expected to have less mucin than that in normal gastric mucosa.

## Figures and Tables

**Table1:** Acute and chronic anisakiasis characteristics

	Acute	Chronic
Symptoms	Severe (abdominal pain, nausea, vomiting)	Asymptomatic or mild (intermittent abdominal pain)
Allergic reaction	Immediate allergy	Not sensitized
Endoscopic findings	*Anisakis* larvae are found on the mucosa, with edema and reddish erosions	Submucosal tumor (*Anisakis* larvae are found in the submucosa)
Pathological findings	Eosinophilic phlegmonous inflammation	Foreign body granuloma

**Table2:** Blood chemistry results

Test item	Measured value	Test item	Measured value
WBC	7.4×10^3^/μL	LD (LDH)	158 U/L
RBC	4.99×10^3^/μL	AMY	116 U/L
PLT	18.4×10^4^/μL	BUN	12.1 mg/dL
Basophil	1%	Cre	0.95 mg/dL
Eosinophil	1%	Na	143 mmol/L
Segmental neutrophil	60%	K	3.7 mmol/L
Lymphocyte	35%	Cl	107 mmol/L
Monocyte	4%	CRP	>0.3 mg/dL
AST	17 U/L	CEA	1.4 ng/mL
ALT	13 U/L	CA19-9	3.8 U/mL

WBC, white blood cell; RBC, red blood cell; PLT, platelet; AST, aspartate aminotransferase; ALT, alanine aminotransferase; LDH, lactate dehydrogenase; AMY, amylase; BUN, blood urea nitrogen; Cre, creatinine; Na, sodium; K, postassium; Cl, chloride; CRP, C-reactive protein; CEA, carcinoembryonic antigen; CA19-9, carbohydrate antigen 19-9.

**Table3:** Previous reports of cases of coexistent cancer and anisakiasis

Year	Age	Sex	Organ of cancer	Organ of anisakiasis	Attached/ Detached	Clinical diagnosis (gross findings/clinical diagnosis of cancer depth)	Pathological diagnosis of cancer depth	Comparison of clinical and pathological diagnosis	Cancer histology	pStage	Method of resection	Author	Reference
1966	42	M	stomach	stomach	detached	early III+IIc	m		sig	IA	subtotal gastrectomy	Hara	48
1970	68	M	stomach	stomach	attached	early IIa	sm		tub2	IA	subtotal gastrectomy	Hayakawa	12
1971	41	M	stomach	stomach	attached	benign ulcer	ss	underdiagnosis	tub	IIA	subtotal gastrectomy	Shinohara	13
1973	53	M	stomach	stomach	attached	early IIa	m		tub	IA	subtotal gastrectomy	Nakajima	14
1973	41	M	stomach	stomach	detached	—	—		—	—	—	Ogata	15
1980	72	M	stomach	stomach	attached	submucosal tumor	sm	underdiagnosis	tub1	IA	subtotal gastrectomy	Shinohara	16
1983	73	M	stomach	stomach	attached	early IIc	m		tub1	IA	subtotal gastrectomy	Tsutsumi	6
1987	48	M	stomach	stomach	attached	advanced type III	se		sig	IIIB	subtotal gastrectomy	Shinoda	17
1988	34	M	stomach	stomach	attached	early IIc	m		sig	IA	total gastrectomy	Hata	18
1988	45	M	duodenum	duodenum	attached	duodenal ulcer with perforation	ss	underdiagnosis	sig	IIA	subtotal gastrectomy	Hirata	19
1988	—	—	stomach	stomach	detached	—	—		—	—	gastrectomy	Yazaki	20
1991	—	—	stomach	stomach	attached	early IIc	—		—	—	—	Kitamura	21
1991	64	F	stomach	stomach	detached	early IIc+ submucosal tumor	sm		sig>por, tub1-tub2	IA	gastrectomy	Masuda	22
1991	65	M	stomach	lymph node	detached	—	—		—	—	total gastrectomy	Yazaki	23
1992	46	M	stomach	lymph node, jejunum	detached	early IIc and metastasis of lymph node and jejunum	—	overdiagnosis	—	—	total gastrectomy/partial resection of jejunum	Hashiguchi	24
1992	59	M	stomach	lymph node	detached	early IIc+IIa	m		tub1	IA	distal gastrectomy	Nishikawa	25
1992	41	M	stomach	stomach	attached	advanced type I	sm	overdiagnosis	pap>medullary with producing AFP	IA	total gastrectomy	Shirasaki	26
1996	65	M	stomach	peritoneum	detached	advanced type I and dissemination of peritoneum	ss	overdiagnosis	pap	IIA	total gastrectomy	Tochika	27
1999	71	M	stomach	stomach	attached	early IIc	m or sm (early)		tub2	IA	distal gastrectomy	Nishioka	28
2000	53	F	stomach	stomach	attached	advanced type II	ss		tub1	IIB	distal gastrectomy	Kuramochi	29
2000	43	F	stomach	stomach	attached	—	m or sm (early)		tub1	IA	subtotal gastrectomy	Maggi	30
2000	34	F	ascending colon	lymph node	detached	metastasis of lymph node	—	overdiagnosis	tub2	—	right hemicolectomy	Maggi	30
2003	61	F	stomach	lymph node, omentum	detached	early IIb and metastasis of lymph node and omentum	m	overdiagnosis	sig	IA	distal gastrectomy	Sakurai	31
2004	56	M	stomach	lymph node	detached	early IIc	m or sm (early)		tub1	IA	distal gastrectomy	Ohishi	32
2004	77	M	stomach	stomach	attached	early IIc or advanced type III	m or sm (early)	overdiagnosis	pap	IA	distal gastrectomy	Saitoh	33
2004	58	M	stomach	stomach	attached	advanced (ss)	ss		por	IIB	distal gastrectomy	Shibahara	34
2005	53	M	stomach	stomach	detached	early IIc	—		—	—	—	Kato	35
2005	61	M	stomach	stomach	detached	early IIa+IIc	m		sig	IA	proximal gastrectomy	Meguro	36
2006	69	M	ascending colon	ascending colon	attached	advanced	ss		tub1	—	right hemicolectomy	Mineta	5
2007	71	M	stomach	transverse colon	detached	advanced gastric cancer (mp) and colonic submucosal tumor	mp		tub1	IB	distal gastrectomy	Satoh	37
2007	68	M	stomach	stomach	detached	—	—		tub1	—	—	Sunakawa	38
2008	50	F	sigmoid colon	ascending colon	detached	double primary cancer	ss	overdiagnosis	—	IIA	total colectomy	Yoo	39
2009	72	F	stomach	stomach	attached	early IIc	m		tub1	IA	endoscopic submucosal dissection	Ohira	40
2012	45	F	sigmoid colon	lymph node	detached	metastasis to lymph nodes	ss	overdiagnosis	tub2	IV	sigmoidectomy	Hernandez-Prera	41
2012	70	F	ascending colon	ascending colon	attached	early (sm)	sm		pap>por1	IA	ileocolic resection	Hiramoto	42
2015	63	M	stomach	stomach	attached	early IIc	sm		sig	IA	distal gastrectomy	Sonoda	11
2018	54	M	stomach	stomach	attached	advanced (se)	mp	overdiagnosis	tub1, tub2	IB	total gastrectomy	Nakagawa	43
2020	70s	M	stomach	stomach	attached	early IIb	m		tub2>por2, sig	—	endoscopic submucosal dissection	Soma	44
2020	40s	F	stomach	stomach	attached	early IIb	m		sig	IA	endoscopic submucosal dissection	Sugai	45
2021	54	M	stomach	stomach	attached	advanced (ss)	mp	overdiagnosis	—	IB	total resection	Nonogaki	46
2021	80s	M	stomach	stomach	attached	advanced (mp)	sm	overdiagnosis	por1>tub2>tub1	IA	distal gastrectomy	Sai	47
2022	40	M	stomach	stomach	attached	early (sm)	m	overdiagnosis	tub1	IA	distal gastrectomy	Sakurai	our case

*Abbr*: m, mucosa; sm, submucosa; mp, muscularis propria; ss, subserosa; se, serosa exposed; tub1, well differentiated tubular adenocarcinoma; tub2, moderately differentiated tubular adenocarcinoma; pap, papillary adenocarcinoma; por1, poorly differentiated adenocarcinoma, solid type; por2, poorly differentiated adenocarcinoma, non-solid type; sig, signet-ring cell carcinoma. This table is modified from the article by Sonoda et al. (ref. 11).
